# Force Sensitive Handles and Capacitive Touch Sensor for Driving a Flexible Haptic-Based Immersive System

**DOI:** 10.3390/s131013487

**Published:** 2013-10-09

**Authors:** Mario Covarrubias, Monica Bordegoni, Umberto Cugini

**Affiliations:** Dipartimento di Meccanica, Politecnico di Milano, via La Masa 1, 20156 Milano, Italy; E-Mails: monica.bordegoni@polimi.it (M.B.); umberto.cugini@polimi.it (U.C.)

**Keywords:** flexible sensor, conformable sensor, tactile data processing, haptic interface, haptic strip

## Abstract

In this article, we present an approach that uses both two force sensitive handles (FSH) and a flexible capacitive touch sensor (FCTS) to drive a haptic-based immersive system. The immersive system has been developed as part of a multimodal interface for product design. The haptic interface consists of a strip that can be used by product designers to evaluate the quality of a 3D virtual shape by using touch, vision and hearing and, also, to interactively change the shape of the virtual object. Specifically, the user interacts with the FSH to move the virtual object and to appropriately position the haptic interface for retrieving the six degrees of freedom required for both manipulation and modification modalities. The FCTS allows the system to track the movement and position of the user's fingers on the strip, which is used for rendering visual and sound feedback. Two evaluation experiments are described, which involve both the evaluation and the modification of a 3D shape. Results show that the use of the haptic strip for the evaluation of aesthetic shapes is effective and supports product designers in the appreciation of the aesthetic qualities of the shape.

## 1. Introduction

The haptic tool presented in this paper, consisting of a continuous servo-controlled physical strip, aims to represent a step forward in the field of digital surfaces manipulation. Such device allows a continuous, free hand contact on a developable strip bent and twisted by a modular servo-controlled mechanism and is integrated with a multimodal, virtual reality system. The objective of the system is to experiment a new tool that allows industrial designers and stylists to perform an effective assessment of the aesthetic quality of the shape of new products and also its modification, directly on the digital prototype, in an intuitive, natural and easy manner, without the need to construct a physical prototype.

The motivation inspiring this research is the following. Current digital tools for shape creation and evaluation (Computer Aided Design—CAD tools), nowadays largely used for the design of products with aesthetic value, are too technical for industrial designers. Therefore, we have developed an interface and an interaction modality that allows the designers, who are generally more keen on expressing their ideas using their own hands to craft physical prototypes, to manipulate a digital prototype by mimicking the operations physically made in reality. The system developed is based on a haptic device based on the concept of a bendable strip; it permits six degrees of freedom (6-DOF) translation and orientation of a digital shape and haptic evaluation of the shape along user-selected geodesic trajectories.

The haptic interface has been described in detail in [1,2]. This paper focuses on the description of the components of the haptic interface (a bendable strip) allowing users to manipulate and apply deformations to the digital shape. Specifically, the strip can be used in three different modalities: to manipulate (move and rotate) the virtual object, to haptically render a curve on the virtual object and to apply global and local deformations to the virtual object. The shape manipulation and deformation are performed by interpreting the user's intent during his interaction with the strip. The deformation applied is computed through the integration of information coming from the force-sensitive handles positioned at the extremities of the strip and the capacitive touch sensor placed along its length. The full system functionalities are controlled through four buttons integrated at the strip extremities and used for the selection of the items of a menu.

The paper presents the system specification, the kinematic mechanism of the haptic interface, the sensors integrated and an example of the use of the device operated by a designer for the exploration and modification of the virtual shapes of some products.

## 2. Related Work

Haptic devices are divided in two major categories: force-feedback devices that allow a point or multi-point contact with virtual objects [3] and tactile displays that can reproduce the shape of a surface, but still of very limited dimensions [4]. Several research works in the field of haptics have addressed the problem of realistically representing curves and curvature information, overcoming the limits of the point-based devices and also providing cutaneous information to fingers. In [5], an approach is presented to give the illusion of a haptic shape only through the communication of the local tangency of the curve on one or more fingertips. This device does not provide enough kinesthetic cues, especially for large curves. Frisoli *et al.* in [6] describe a haptic device that is the combination of a point-based device to provide kinesthetic cues and a fingertip haptic device that provides cutaneous cues. In [7] is described an attempt to communicate curves and curvatures through a contact location feedback on the fingertips. The main limitation of these three systems is that users interact with the shape using only a part of the hand, mainly one or more fingers, and not the whole hand.

Some research activities have addressed the limits of human perception and discrimination of curvature in a whole-hand exploration, like those reported in [8,9]. The haptic strip we have developed is an attempt at developing a haptic interface that tries to reproduce exactly the shape of a curve, by deforming a physical continuous strip, in order to give users the possibility to perform a full and full-hand contact with the virtual surface. Therefore, the haptic strip we have developed is an interface that is able to communicate both tactile and kinesthetic cues through a whole hand interaction.

Some of the most relevant force feedback technologies are the following [10]: point-based devices, like the PHANToM [11], the MOOG-HapticMaster [12] and the Haption-Virtuose [13], and multi-point based devices, like the Haptex [14] system. Some interesting research works related to surface physical rendering are based on vertical pin displacement for mid-scale virtual surfaces [15] or, more recently, on a miniature pin array tactile module [16] based on elastic and electromagnetic forces for mobile devices, which provides enough working frequency, output force and amplitude to stimulate the human's mechanoreceptors. A small and lightweight tactile display described in [17] is integrated into a haptic glove system. In [18] is presented a tactile display using airborne ultrasound: the prototype presented provides weak force for users to feel constant pressure, just sufficient for vibratory sensation. In [19] is described a continuous tangible user interface for modeling freeform 3D objects, such as landscape models, by scanning and illuminating a clay or a SandScape; nevertheless, the system does not provide a force feedback. There are several studies using the Immersion CyberGlove [20], whose major drawback is that it is quite invasive, because the user basically needs to wear a glove covered by an exoskeleton. In general, the drawback of wearable systems is that since the weight and size of the devices are a concern, the systems will have a more limited set of capabilities [21,22].

The haptic interface, developed in the context of the Sound And Tangible Interfaces for Novel product design (SATIN) project [23], has been described in detail in previous papers and consists of a flexible strip that is held in space, in front of the user, by two HapticMaster systems [24]. This setup allows for six degrees of freedom in the movement of the haptic strip. The strip consists of a series of nine equidistant relative actuators, which allow the strip to actively shape itself in order to match the digital object along a selected geodesic curve. The strip is inspired by the deformable tape that designers use for creating and modifying aesthetic physical shapes. The first version of the haptic strip has been developed with the main objective of integrating the various mechanical components in order to validate the concept at its basis, which is related to the cutting plane metaphor. Subsequently, a better performing version of the haptic strip has been designed, with the aim of extending the domain of curves that can be haptically rendered [25]. This second version of the strip is capable of rendering geodesic trajectories in addition to planar ones. The mechanical configuration of both versions of the strip allows us to reproduce curves that lie on the virtual object, but they both achieve a minimum-bending radius of 180 mm, which directly limits the total curves that the haptic strip is able to represent. Obviously, the smaller the bending radius of the haptic strip, the broader is the domain of virtual shapes that is possible to render.

## 3. System Description

The deformable haptic strip is a part of a multimodal system conceived for the evaluation and the modification of digital shapes [26]. The aim of the research is to mimic assessment operations performed by designers on real prototypes using physical splines. Similarly, the haptic strip is laid over the surface of the stereoscopic visualization of a virtual shape.

The strip has been designed so as to provide several interaction modalities with the rendered virtual object. It allows: (1) the 6-DOF positioning of the virtual object within the workspace; (2) the haptic rendering of user-defined curves belonging to the surface of the virtual object; (3) the inspection of each selected curve by touching the haptic strip; (4) during the curve inspection, the hearing of the sound associated with the geometric characteristics of the curve, like the curvature function, inflection points and discontinuities; and finally (5) the local and global deformation of the virtual shape using the force-sensitive handles at the extremities of the strip.

A structure mounting a Digital Light Processing (DLP) projector and equipped with a semi-reflective mirror is able to provide 3D stereoscopic visualization, head tracking, stereo sound system and a co-located perception of the virtual model and the haptic strip, allowing the user to perceive the strip exactly where it is projected within the virtual workspace [1]. The users wear shutter stereo glasses in order to see in stereoscopy.

### 3.1. Shape Manipulation Based on Capturing User's Intent

The shape manipulation and modification is based on the concept of capturing the user's intent. Basically, the idea is trying to predict the intention that the user has of manipulating and deforming the shape, on the basis of the actions performed on the strip extremities, and apply a coherent repositioning and reshaping of the virtual object.

**Figure 1.**
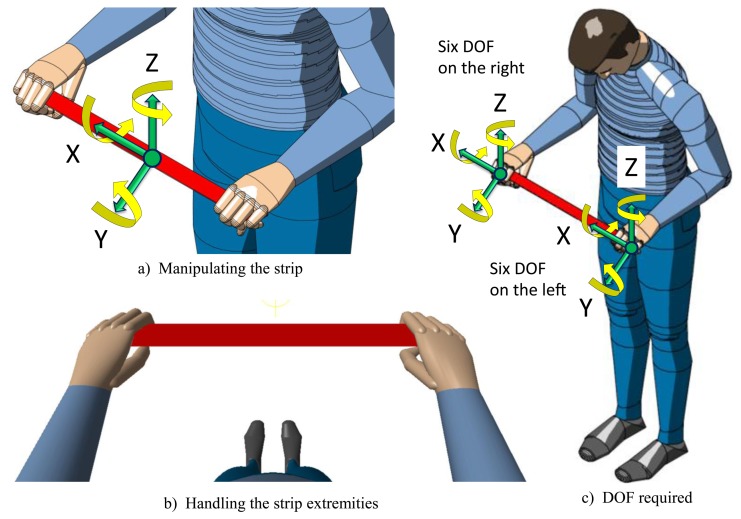
User's intent for manipulating the haptic strip.

The concept of the haptic strip for capturing the user's intent is shown in Figure 1. The haptic strip is correlated with the virtual shape by anchoring the coordinate system located on the center point of the strip with the virtual object (Figure 1a). When the user moves the haptic strip, the virtual object, anchored to it, is moved accordingly (Figure 1b).

A system with six degrees of freedom is required to correctly manipulate the virtual object. Therefore, the strip is equipped with control devices positioned at the extremities, consisting of optical or mechanical sensors, as can be seen in Figure 1c. Their objective is capturing the user's intent of manipulating and deforming the shape by means of detecting six degrees of freedom on each device. The two devices, one at each extremity, are used for manipulating the haptic strip with both hands. By manipulating the strip extremities, the users can apply a local deformation, applied in a specific point of the strip, or a global deformation, applied to all of the curve and, consequently, possibly affecting all of the shape.

## 4. Force Sensitive Handles (FSH)

**Figure 2.**
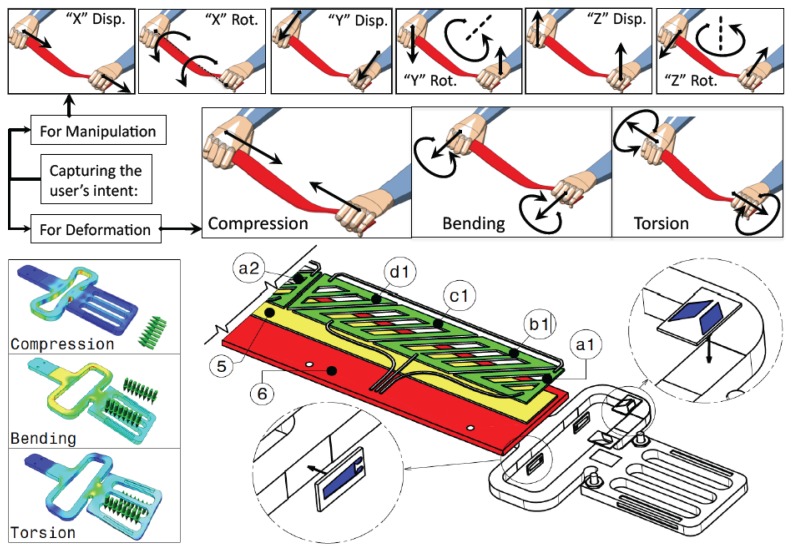
Force sensitive handles (FSH) configuration.

The strip can be used in three different modalities: to manipulate (move and rotate) the virtual object, to haptically render a curve on the virtual object and to apply global and local deformations to the virtual object. That means that the user has to handle the strip somehow for both manipulating and applying deformations to the strip. The first row in Figure 2 shows the six degrees of freedom required for manipulating the haptic strip as a 6-DOF platform. The second row shows the user's intent action for modification (compression, bending and torsion).

The handling happens through the strip extremities, which are sensorized. A solution based on strain gauge sensor technology has been analyzed in order to check the capability of retrieving the degrees of freedom required for both shape manipulation and modification.

A strain gauge is a sensor whose resistance varies with the applied force. It converts force, pressure, tension, weight, *etc.*, into a change in electrical resistance, which can then be measured. These sensors are used for measuring strain (tensile and compressive strain, distinguished by positive or negative sign). The 1-LY13-6/350 and 1-XY43-3/350 strain gauge models provided by HBM (Hottinger Baldwin Messtechnik) [27] have been installed into the strip, following certified procedures.

The shape of the FSH at the strip extremities and the locations and characteristics of strain gauges to be used have been designed and verified through structural analysis of the user's conditions, before its physical implementation.

## 5. Flexible Capacitive Touch Sensor (FCTS) and Lighting

The sound interface allows the sonification of some characteristics of the curve. In particular, the system plays metaphoric sounds associated with curve characteristics during the user's exploration of the haptic strip. Curve sonification has been introduced because of two reasons: first, because it is effective for rendering information about a curve through multiple modalities, which are not only vision; second, because due to the limits of the strip, some characteristics of the curve, e.g., curvature discontinuities, cannot be haptically rendered. Sound can be an effective alternative in this sense.

Therefore, we have introduced metaphoric sounds that can be played according to the kind of local geometric characteristics of the curve. To do that, it is necessary to track the user's finger position on the strip surface.

**Figure 3.**
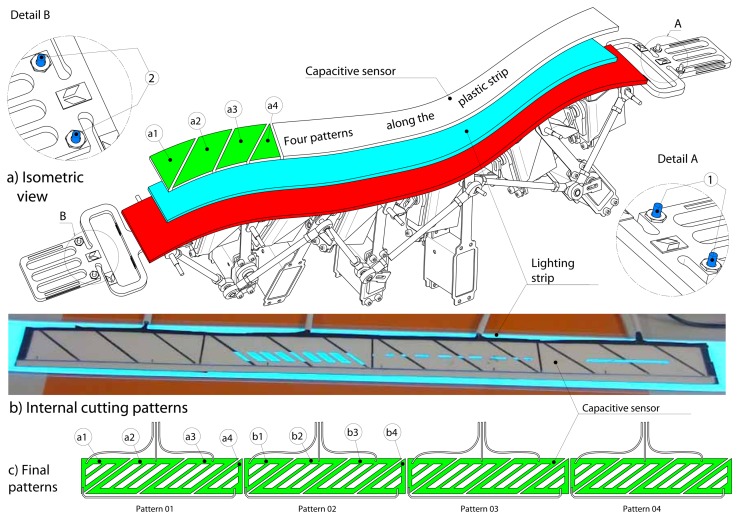
Flexible capacitive touch sensor (FCTS).

We have analyzed some technical solutions for tracking the position of the user's fingers. A possibility initially investigated was using data gloves. Actually, this solution is invasive and seems to limit the freedom of designers. An alternative feasible solution proposed was using tactile sensors. Using a flexible capacitive touch sensor (FCTS) is an effective solution, because the user does not need to wear any device, nor to apply any pressure to activate the sensorized surface of the strip. In fact, the sensor is easily activated through a light contact. Figure 3a shows the FCTS positioned on top of the strip.

As shown in Figure 3b, different patterns have been analyzed. An electric field is formed between the receiver and the transmitter trace. We used four QProx E1101 development boards, which provide excitation to the capacitance sensor, sense the changes in capacitance caused by the user's proximity and provide a digital output. The final pattern configuration is displayed in Figure 3c. The first board uses the metal traces, a1, a2, a3 and a4, to track the user's finger; the second board uses the metal traces from b1 to b4, and so on. In this way, we have designed a flexible multi-touch strip.

The detection of the position of the user's finger on the strip is also useful for allowing a modification of the shape, which can be performed at the point where a local force is applied.

### 5.1. Lighting

The FCTS also includes a lighting system in order to better visualize the physical strip in the 3D stereo visualization system. While the users interact with the virtual object through the haptic strip, it is necessary to see where the hands are positioned in space, not only to appropriately handle the force sensitive extremities, but also to appreciate the co-location with the virtual object. To illuminate the haptic strip, we have considered two options: fluorescent and phosphorescent materials glued on top of the plastic strip.

We have made some user tests for both the solutions. In both cases, the user was not able to ‘see’ the fluorescent/phosphorescent strip. In fact, the light reflected using the fluorescent/phosphorescent strip was not enough to pass through the half-silvered mirror and the shutter glasses worn by the users. In other words, we realized that the light has to be generated by the strip and not only reflected. Therefore, we decided to use lighting strip technology [28] that is usable for great lighting effects, as well as a very appealing and flexible backlit display. The lighting strip is driven by an AC inverter, which depends on the total light surface of the lamp. Of course, high surfaces can be lit, but the limit is set by the size of the drivers available. Since the strip generates an electric field that interferes with the electric field of the capacitive sensor used for finger tracking, it has been positioned under the capacitive sensor, as can be seen in Figure 3a. This array allows us to see a continuous light of 10 mm width along the strip length.

## 6. Kinematic Mechanism

**Figure 4.**
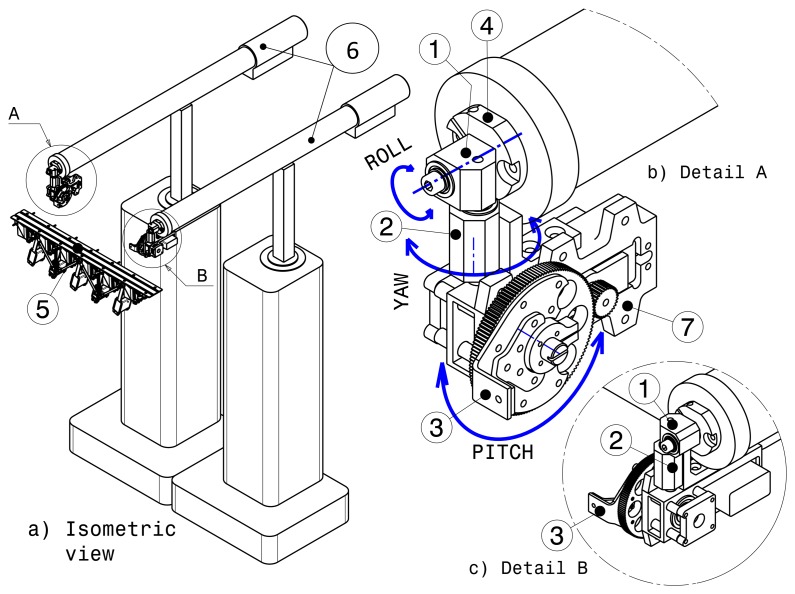
Final concept of the 6-DOF platform.

Several possible configurations have been analyzed in order to provide tilting and the components needed to clamp the haptic strip mechanism on the two MOOG-HapticMaster (HM) devices. Figure 4 shows the final concept in which is used a reduction gear mechanism mounted on each end effector of the MOOG-HM devices.

Using the reduction gear for tilting is a novel solution. It simplifies the inverse kinematics needed to control the 6-DOF platform. However, balancing the whole strip mechanism has been necessary, because the servos are not infinitely strong. On the other hand, we do not use mechanical links that produce a low relationship between the two HM devices and the platform, as in the two previous concepts. With the reduction gear actuator, we dispense with the straight link between the two HM devices altogether and use the extra link to accommodate both the distance variation between the HM devices and the length variation between the interpolation points.

As shown in Figure 4, the geodesic mechanism (5) is attached on the two HM devices (6), through the sheet metal component (3) mounted on the tilt mechanism (7). Three components have been required to connect each end effector of the HM device with the geodesic mechanism. The first one is the roll component (1). The second one is the yaw component (2), and the last is the tilt mechanism (7). The geodesic mechanism is clamped and unclamped using a quick clamping device (4). Using this solution, five degrees of freedom in the positioning of the strip (3 DOF translation, roll and yaw) are provided by the HM devices, and pitch is provided by two gear reduction systems.

**Figure 5.**
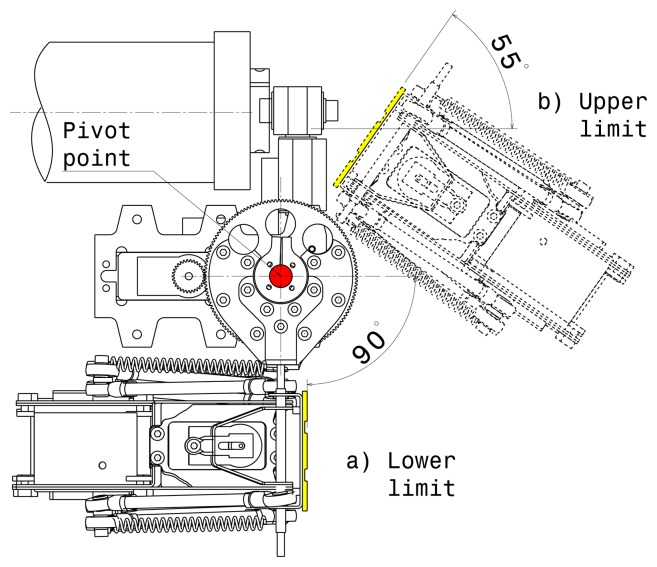
Limits in pitch degree of freedom.

This configuration provides five degrees of force feedback (during strip positioning, roll and yaw). Using two gear reduction systems to control the tilt permits direct and stiff connection between the HM devices. The servo drives have been selected so as to guarantee high reliability: the servo motor with titanium gears provides up to 2.35 Nm of continuous torque, and the gear reduction systems 5:1 are HS-5955TG manufactured by HITEC. This allows us to get high stiffness and load capacity even when the user is applying pressure while exploring the strip. Figure 4c shows the components mounted on the right end effector of the MOOG-HM devices. This configuration is practically a mirror of the components mounted on the left end effector. While tilting, the geodesic mechanism is able to rotate from −55 to +90 degrees in the ‘X’ axis, as can be seen in Figure 5. Regarding the yaw limits, the geodesic mechanism is able to reach ±62 degrees in the ‘Z’ axis without any collision. With regards to the roll limits, the geodesic mechanism rotates from +164 to −164 degrees around the ‘Y’ axis.

The positioning of the strip is controlled by defining the target position of the end point of each MOOG-HM device and the value of the pitch angle (controlled by digital servos).

## 7. Use of the Haptic Strip

The following pictures show the haptic strip as it has been implemented according to the specification and technical solutions presented in the previous sections.

**Figure 6.**
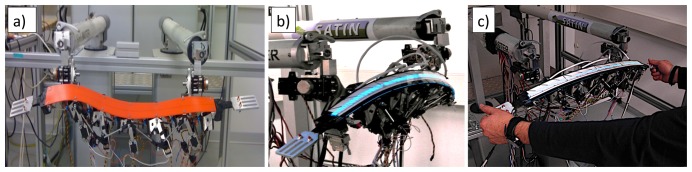
Aluminum sensorized extremities mounted on the haptic strip.

Figure 6a shows the aluminum sensorized extremities mounted on the haptic strip, one on the left- and one on the right-hand side of the strip, without finger tracking and lighting strip systems.

Figure 6b shows the haptic strip mechanism with all its main components: the mechanical links in which it lies, the sensorized extremities, the user's fingers tracking system, the components used to clamp the haptic strip on the two MOOG-HapticMaster devices and the lighting strip.

Figure 6c shows the way in which the user is handling the sensorized extremities of the haptic strip for both manipulation and modification modalities.

The haptic interface that we have developed provides three main interaction modalities, which are described in the following sections.

### 7.1. Exploration Modality

**Figure 7.**
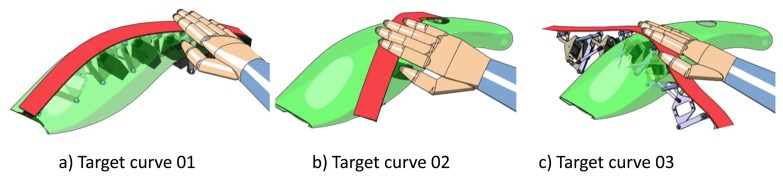
Different target curves during the curve exploration.

When the exploration modality is active, the haptic strip remains static, allowing a stable evaluation of the curve represented by the haptic strip. Let us consider the case in which the user has positioned the haptic strip, as can be seen in Figure 7a, by means of the two sensorized extremities. Once the target position is reached, the user is able to freely explore and feel the shape of the virtual object by using his dominant hand. This operation is performed in the same way as in the real world.

In the exploration modality, the virtual object remains fixed in space, and the haptic strip slides on the virtual object surface. Figure 7b shows the case in which the haptic strip has been rotated on the ‘Z’ axis; during this operation, the strip conforms according to the cutting plane metaphor in real-time. Figure 7c shows the limit in the yaw angle of the haptic strip as a 6-DOF platform.

While moving the sensorized extremities, the haptic strip continuously conforms to the surface. By moving the strip, the user manipulates only the cutting plane, so the displacement motion has the following degrees of freedom:

1. Forward/backward translation: moves virtual plane
2. Rotating in top view: rotates virtual plane
3. Tilting backward and forward: rotates virtual plane
4. Sliding to left or right: moves virtual plane

The exploration modality also offers the possibility to use the curvature data of models physically represented by the strip in order to render sound. The sound gives acoustic information about inflexion points, discontinuities and curvature.

**Figure 8.**
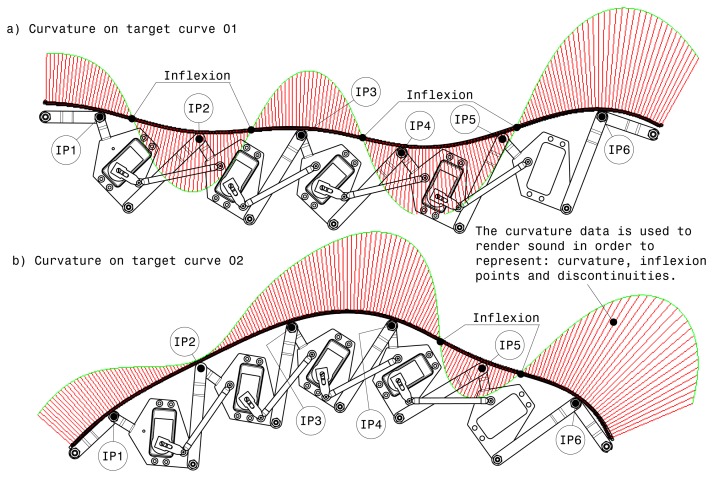
Examples of various curvatures.

Figure 8 shows two target curves in which some curve properties are displayed. As can be seen in Figure 8a, the curve has four inflexion points that are precisely communicated and georeferenced to the user by means of sound. Sonification is effectively used to convey the variation of local curvature during the curve exploration. In the case of curvature discontinuity in the virtual model, because the plastic strip is a continuous surface and cannot provide this information haptically, sounds can convey the information about the precise localization of the curvature discontinuity. Furthermore, the sound metaphor is used in order to show the correct position of the curve properties through the visualization system. While the user slides the hand on the real haptic strip, the capacitive sensor mounted on the strip tracks the hand position and renders its position in real-time. When the hand is on a discontinuity point or at an inflexion point, the auditory module activates a characteristic sound that represents those points. Figure 8b shows the target curve 02 in which are represented two inflexion points. When the user hand is positioned before interpolation point 5 (**IP5**), and near the first inflexion point, the position of this inflexion point is rendered through sound. The same procedure is used to render by sound the second inflexion point.

### 7.2. Modification Modality

Two options have been implemented for the modification modality, consisting of global and local bending on the curve controlling the shape of the virtual object. The two kinds of modification modalities are described in the following sections.

#### 7.2.1. Global Modification

While the virtual object is deformed, the visual representation of the model is modified as well, and the strip description is updated.

The modification modality can be switched off by selecting a button. The user can also undo or apply the modification at any time by using the small buttons located at the extremities of the trip. The undo command recovers the previous state of the model and exits from the modification state. By applying the modification, the deformation is replicated to all existing CAD surfaces and CAD curves of the model, and finally, the application exits from the modification state. During the interactive modification, only the visual representation of the model is deformed instead of the CAD surfaces. This is done in order to offer a real-time feedback of the effect of reliable interactive deformation.

**Figure 9.**
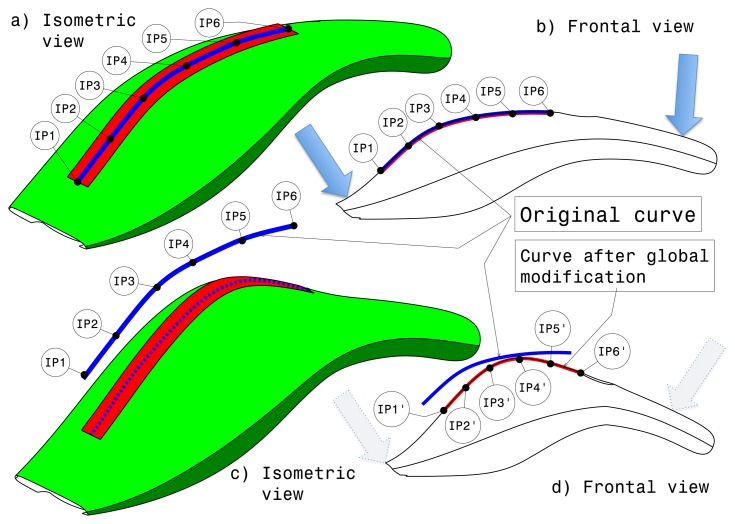
Global modification.

Figure 9a shows the isometric view of the virtual object before the application of the global modification. The haptic strip is represented as a red surface on the object, and the blue curve is the curve in the middle of the haptic strip, displaying the six interpolation points. Figure 9b shows the frontal view in which is displayed the original curve represented by the haptic strip. As mentioned before, when the global modification modality is active, the user is able to deform globally the virtual object by using the strip extremities.

By using the force sensitive handles, the user is able to bend the virtual object, as can be seen in Figure 9c; the blue continuous curve represents the original curve, and the blue dashed one represents the curve after applying the global modification. Figure 9d shows the position of the new interpolation points.

#### 7.2.2. Local Modification

**Figure 10.**
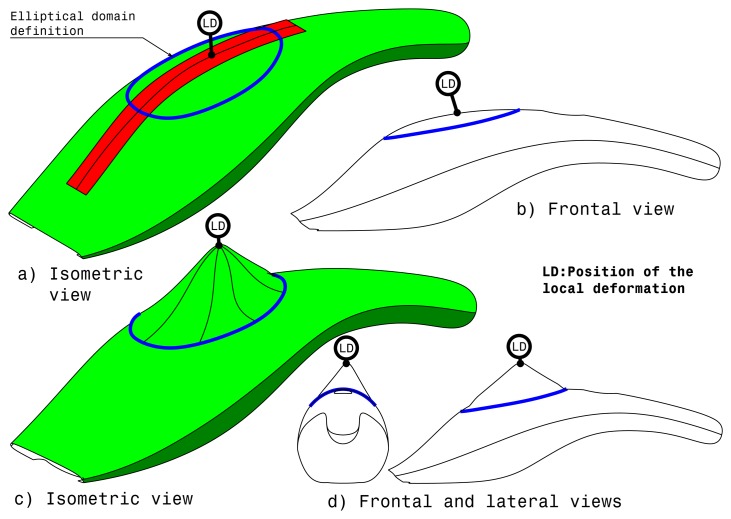
Local modification.

The local modification relies on a deformation applied by interacting with the haptic strip and requires the selection of a target curve. Differently from the previous method, the local modification allows the user to perform a more controlled deformation by defining two new control attributes: the domain and the location of the point where the deformation is locally applied by pushing or pulling on the strip, as can be seen in Figure 10a.

The local modification domain is an area around the strip curve that represents the boundaries of the influence of the local deformation. The domain is used to identify which surfaces of the model are affected by the deformation. This domain is represented as an ellipsoid surface. The ellipse is displayed in a plane that is perpendicular to an average normal to the surface along the strip; then, this ellipse is projected on the shape to really define the surface modified domain. Then, it is possible to define an additional constraint to be fulfilled along the boundary of the domain. The point on the curve defines the position in which the local modification is applied. This point is located along the curve and can be specified by the user. By applying forces on the haptic strip, the user is able to push or pull the surface on that point using as deformation device the force sensitive handles. Like for the domain boundary, different types of constraints can be applied to better define the deformation. For example, the deformation problem can consider matching the position and the tangency as the targets.

Complete list of local modification attributes:

- Ellipsoid domain
- Constraints on the domain boundary (keep position, keep position + tangency, keep position + tangency + curvature)
- Location of the deformation point along the strip
- Constraint of the local modification (target position, target position + keep tangency, target position + keep tangency + keep curvature). The target position is derived from the initial point where a pression on the strip is applied.

Figure 10a shows the isometric view of the virtual object before local modification. At this point, the user is able to modify the dimension of the blue elliptical surface of influence through the force sensitive handles. This surface of influence can be smaller or larger, so as to satisfy the user requirements. Once the user has defined the blue elliptical surface dimension, it is possible to decide the position of the local modification point, as can be seen in Figure 10b, so the user can apply the local deformation of the virtual object using the force sensitive handles as modification device. Figure 10c,d show the object after local modification.

## 8. Users' Test

Some users' tests have been performed during the evolution of the project in order to assess the conceptual design and to validate the system. In fact, according to a user-centered approach, the evaluation of an interactive system has to be performed several times during the system development in order to ensure that usability issues have been properly addressed in the development [29].

The users involved in the tests were Computer Aided Styling (CAS) designers and model makers working for the companies' partners in the research project; the tests were performed with the collaboration of human factor experts.

### 8.1. Evaluation Protocol

In order to correctly proceed with the test, we have first developed an evaluation protocol. Firstly, participants were given a copy of a short user manual to read prior to commencement of the evaluations. This manual provides a brief overview of the user interface and an introduction to the immersive system and its potential uses. Second, participants have been introduced to the actual system, and finally, participants have been instructed through its operation. This protocol has been applied for enabling users to familiarize themselves with the system.

The evaluation comprised the following main stages:

- *Pre-evaluation questionnaires*. Demographic information has been obtained and consent forms completed for all participants.
- *Pre-evaluation explanation of the system*. A demonstration of the following has been provided: an overview of the system components, an explanation of what the user needed to wear (active stereo glasses), the system in operation and possible tasks that can be performed using the system.
- *During evaluation observations*. Participants have been observed for errors, incidences of asking for help, expressions of frustration, indications that they were experiencing sickness symptoms.
- *Post-evaluation questionnaires*. Information has been gathered of participants' opinions and experiences of using the system, acquiring information about possible improvements and usability.

### 8.2. Evaluation Tasks

Initially, all participants were asked to carry out a number of tasks, which involved using a combination of visual, haptic and auditory functions in order to carry out manipulation, explorations and modifications to a set of models of objects that have been loaded in sequence onto the visualization system. In the tests described hereafter, two different product models have been selected and supplied by the industrial partners participating in the research project: a model of a vacuum cleaner used for the exploration and global modification, a glass vase used for global modification and a car bonnet used for the local modification.

For the *manipulation task*, the model of a vacuum cleaner has been loaded. Then, the two sensorized extremities of the strip have been used to translate and rotate the virtual object.

**Figure 11.**
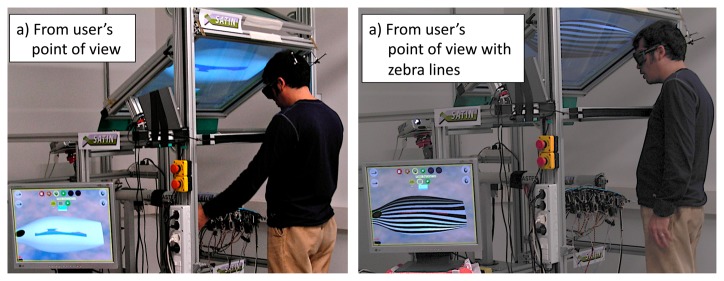
Virtual object seen from the user's point of view.

Figure 11a shows the vacuum cleaner model from the user's point of view; to note on the left bottom corner the PC-monitor that we have used in order to show the correct perspective of the virtual object as seen by the user. Figure 11b shows the vacuum cleaner model in which is enabled the option to render the zebra lines for surface evaluation.

**Figure 12.**
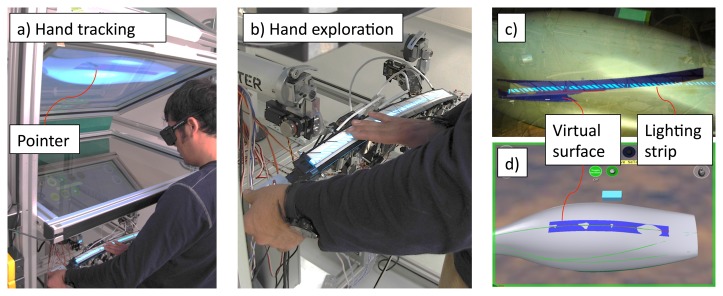
Exploring the virtual object.

For the *exploration* task, users were required to explore the shape along a given curve on the 3D model and to determine both the points of maximum and minimum curvature and the inflexion point(s). Figure 12a shows the user while exploring the virtual vacuum cleaner.

When the user is exploring the virtual surface through the haptic strip, an arrow pointer is displayed by means of tracking the user's hand. This arrow helps the user to understand where exactly the hand is positioned in space. Figure 12c,d show the user's point of view. Note the lighting strip that has been previously included. It allows the user to see the shadow of his hand, so enabling the possibility to easily recognize the position of the real haptic strip.

**Figure 13.**
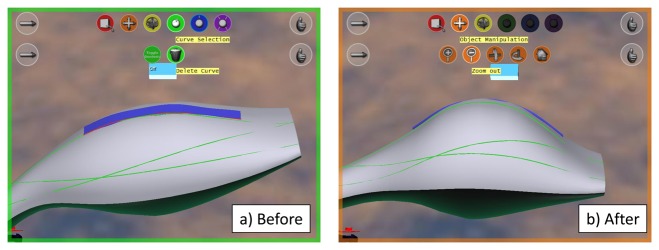
Global modification task performed an a vacuum-cleaner.

For the *global modification* task, each participant was shown ‘before’ and ‘after’ pictures, as shown in Figure 13. The ‘after’ picture depicts the target modification, and the participants were asked to try to apply modifications to the shape, so as to obtain one that resembles the ‘after’ picture.

**Figure 14.**
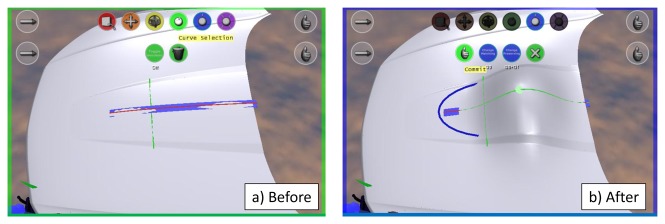
Local modification performed on a car bonnet.

The same procedure has been used for the *local modification* task. ‘Before’ and ‘after’ pictures have been displayed to the users, who were asked to reproduce the modification on the virtual car bonnet. Figure 14 shows the pictures used in this task.

The test has been conducted with 14 participants, who were from the user partner or local engineering and design students recruited. Participants have been selected to be representative of the target user population of the system. Since the aim of the system is for use by modelers, designers and CAD technicians, an attempt has been made to represent all of these groups in the evaluation participant sample. All participants completed the same activities (referred to as ‘tasks’) within the system.

## 9. Results and Discussion

The user's performance has been analyzed in terms of the exploration and modification of objects while using the system. The exploration modality has been assessed in terms of the time taken to detect both the minimum and maximum point of curvature and the time taken to detect an inflexion point(s). The exploration task was completed for the vacuum cleaner model.

Concerning the modification modality, a specific modification task has been assigned to participants for all three object models. The time taken to complete the modification and the number of participants who were successful in task completion has been recorded.

**Figure 15.**
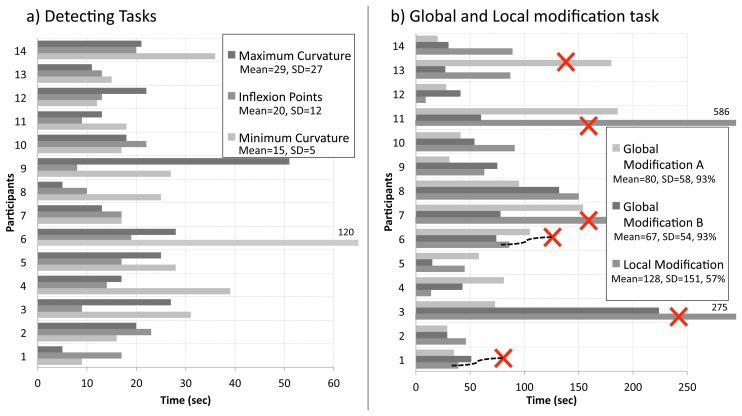
Tasks results.

Chart a on Figure 15 shows the results obtained from the exploration task. The mean time taken to complete the task was highest (29 s) with SD = 27 for the detecting maximum curvature task and lowest for the detecting minimum curvature task (15 s) with SD = 5.

The standard deviation was high for all tasks and, in particular, for the detection of maximum curvature task, illustrating a high level of variation in participant ability (although participant 6's particularly slow performance will have skewed this value).

Chart b on Figure 15 shows the data obtained from the modification elements of the task. Modification A refers to the vacuum-cleaner. Modification B to the glass vase, and local modification refers to the car bonnet. It can be seen that the local modification of the car bonnet was the task that took the longest time, with the global modification of the vase being the fastest task completion. Again, however, there was large variation in participant performance, with one person failing to complete each of the global modification tasks and five failing to complete the local modification (highlighted with a cross in the chart).

This high number of failures to complete the local modification task is of concern. Despite these differences, due to the large standard deviations, there was no significant difference between time taken to complete the three tasks (F = 1.24; df = 2, 12; *p* >0.05).

Whilst the test showed little difference in duration, the success rate for the local modification test was much lower than for the global modification. The main issue in test failure was the ability of the visual system to provide effective feedback. There were two sources of this difficulty. Firstly the viewpoint, where participants were required to look down on the object, was not appropriate for providing of view of the profile of the large object (car bonnet) that would effectively support modification. Secondly, this was exacerbated by the low resolution of the model.

**Figure 16.**
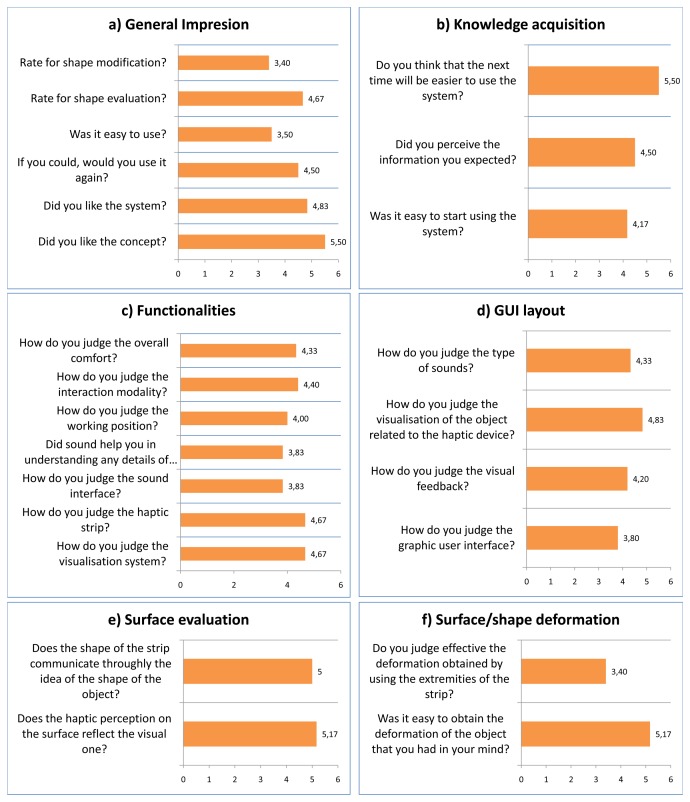
Show case results.

As part of the evaluation of the whole system, we decided to organize some tests, named show cases, with external potential users working in the industrial design field in order to disseminate the concept related to the new technology developed for supporting product design and, also, to collect opinions and feedback about the use of the system. Six testers have been selected, from the industrial design sector or from the virtual reality field. The charts in Figure 16 show the results of this test. The score system proposed has a scale from 1 (most negative value) to 6 (most positive value).

From chart a in Figure 16, it is possible to appreciate that the *general impression* about the system was good. Overall, all the participants reported a high level of appreciation of the concept layout.

The system achieved a high evaluation rate also relative to the aspects concerning the system in general, a quite positive evaluation in the easiness of use as a whole and in using it for evaluating shapes. Specifically, five users out six have judged the system with a rating between five and six, and only one user has evaluated it with a medium score, equal to three. The scores assigned to the effectiveness of the system in evaluating the shapes are comprised between four and five. Still, some problems in terms of effectiveness of the system in modifying the shape came out.

The *knowledge acquisition* part of the questionnaire intended to go more in detail into the understanding and evaluation of the system by the user's perspective. Chart b in Figure 16 shows the results. Overall, the results show a positive evaluation; the users have evaluated this feature by giving scores from four to six. Only one user has assigned a very low rate concerning the easiness of using the system for the first time. However, the same user is convinced that next time, it will be easier to use the system.

As it is possible to observe from chart c in Figure 16, the participants have judged the visualization system and the haptic strip in the *functionalities* aspects of the system as interesting. A positive evaluation has been given also to the interaction modality. The users have stated that they felt an overall comfort, and in general, they have assigned high scores also to the working position. What becomes evident is the fact that they disliked the sound interface, specifically concerning some information provided by it. These results highlight that the general layout and system architecture were coherent and performing.

From the results of the *Graphical User Interface (GUI) layout*, chart d shows that it has been evaluated to be good enough by the users. Furthermore, it seems that some participants were complaining about some functionalities of the system related to aspects inherent in the graphic user interface and the type of sound. Still, the results also show that testers were not so unsatisfied and they have high expectations for future system improvements.

The scores assigned to the two questions related to the *surface evaluation* have been very high, as reported in chart e. Thus, the users have particularly appreciated the possibilities of exploring the surface by means of the haptic strip. Actually, the users have asserted that the perception of the surface reflects the visual one (five users have assigned five scores out of six, and one user has selected the maximum score possible). In addition, the users have recognized the effectiveness of the strip in communicating the idea of the shape of the object that they are seeing thoroughly.

From chart f can be seen the average assessments of the users concerning the functionalities of the *surface/shape deformation*. The users mostly did not really appreciate the precision of the system in following their actions. In particular, the problem was related to the fact that they could not precisely obtain the desired global and local modification.

## 10. Conclusions

The paper has presented a novel haptic interface for rendering and for applying deformations to digital surfaces. The haptic interface consists of a haptic strip that is bent and twisted by a modular servo-controlled mechanism. The strip conforms to a selected curve belonging to a surface and allows users to evaluate the quality of shapes along a line. The strip is also equipped with force sensitive handles placed at its extremities. By acting on the extremities, the user can apply deformations to the digital object shape.

The tests performed with target users, who are industrial designers, have demonstrated that the use of the haptic strip for the evaluation of aesthetic shapes is effective and supports them in the appreciation of the aesthetic qualities of the shape [30]. The use of the strip for the deformation of the digital surface has also been considered an easy and rapid way for prototyping various forms of the digital object. After the participants had completed the evaluation assignments, they were asked to answer a questionnaire about their experience. Detailed information on the specific questions and answers and other human factor aspects of the evaluation can be found in [31]. In this article, we only wished to highlight that participants considered that sound helped them to understand the unseeable curvature information and to better examine the quality of the curve shape.

In general, the users reported a strong appreciation of the concept proposed by the system and the intrinsic possibilities of such technology. In this sense, the result has been that the system has been extremely well conceived, since all characteristics related to the ease of use and the intuitiveness of the system layout were judged extremely positive, as well as all characteristics related to the system components. The users' comments were very valuable in defining where the efforts for increasing our system performances have to be addressed. These results highlight that the general layout and the system's architecture was coherent and performing. The results have clearly highlighted the generation of a correct system layout and the study of multimodal interaction reliable in terms of realism, even for virtual reality and multimodal experts. Some strong problems relative to the user interface and the precision of the haptic strip in modifying the object were persistent. These problems heavily compromised the perception of the system relatively to its quality and potentialities with reference to the modification functionality. Instead, all the users have defined the system as a tool for evaluating the shape and the surfaces of an object as extremely effective. Important results have been the very high evaluation of the concept, as well as the appraisal of the multimodality of the system. Contrary to our expectations, the testers defined the system as being quite ready for being integrated into their daily activity, as well as in industry fields. In contrast with the results gathered in the test evaluation, the sound interface has not been considered good enough. Actually, even if the users have appreciated this way of conveying the information related to the curvature and the inflection points, the users did not consider the sound interface as an effective means for conveying the information related to the surface and the shape. Concluding that the general impressions about the system provided by the limited, but meaningful, number of testers was quite good, and some improvements in terms of user interface and interaction have been suggested as necessary in order to obtain a completely coherent and well performing system.

Further research, however, is still needed to improve the performance of the haptic strip device to meet more demanding industrial applications by using a desktop and portable device and to improve the minimum-bending radius. A first attempt to replicate the concept has been explained in [32].

## References

[1] Bordegoni M., Ferrise F., Covarrubias M., Antolini M. A Linear Interface for the Evaluation of Shapes.

[2] Bordegoni M., Cugini U., Covarrubias M., Antolini M. Geodesic Haptic Device for Surface Rendering.

[3] Hayward V., Ashley O., Hernandez M.C., Grant D., Robles-De-La-Torre G. (2004). Haptic interfaces and devices. Sens. Rev..

[4] Chouvardas V., Miliou A., Hatalis M. (2008). Tactile displays: Overview and recent advances. Displays.

[5] Dostmohamed H., Hayward V. (2005). Trajectory of contact region on the fingerpad gives the illusion of haptic shape. Exp. Brain Res..

[6] Frisoli A., Solazzi M., Salsedo F., Bergamasco M. (2008). A fingertip haptic display for improving curvature discrimination. Presence Teleoperators Virtual Environ..

[7] Provancher W.R., Cutkosky M.R., Kuchenbecker K.J., Niemeyer G. (2005). Contact location display for haptic perception of curvature and object motion. Int. J. Rob. Res..

[8] Pont S.C., Kappers A.M., Koenderink J.J. (1997). Haptic curvature discrimination at several regions of the hand. Percept. Psychophys..

[9] Sanders A.F., Kappers A.M. (2009). A kinematic cue for active haptic shape perception. Brain Res..

[10] Hayward V., Ashley O., Cruz Hernandez M., Grant D., Robles DeLaTorre G. (2004). Haptic interfaces and devices. Sens. Rev..

[11] PHANToM Device. http://www.sensable.com.

[12] FCS-HapticMaster. http://www.moog.com/.

[13] http://www.haption.com/.

[14] Haptex System. http://haptex.miralab.unige.ch/.

[15] Iwata H., Yano H., Nakaizumi F., Kawamura R. Project FEELEX: Adding Haptic Surface to Graphics.

[16] Tae-Heon Y., Sang-Youn K., Chong-Hui K., Dong-Soo K., Wayne J. Development of a Miniature Pin-array Tactile Module Using Elastic and Electromagnetic Force for Mobile Devices.

[17] Kim S.C., Kim C.H., Yang G.H., Yang T.H., Han B.K., Kang S.C., Kwon D.S. Small and Lightweight Tactile Display(SaLT) and Its Application.

[18] Takayuki H., Takayuki I., Hiroyuki S. Non-contact Tactile Sensation Synthesized by Ultrasound Transducers.

[19] Ishii H., Ratti C., Piper B., Wang Y., Biderman A., Ben-Joseph E. (2004). Bringing clay and sand into digital design—Continuous tangible user interfaces. BT Technol. J..

[20] Cyber Glove Systems. http://cyberglovesystems.com/?q=products/cybergrasp/overview/.

[21] Bouzit M., Popescu G., Burdea G., Boian R. The Rutgers Master II-ND Force Feedback Glove.

[22] Bullion C., Gurocak H. (2009). Haptic glove with MR brakes for distributed finger force feedback. Presence Teleoperators Virtual Environ..

[23] Bordegoni M., Ferrise F., Covarrubias M., Antolini M. (2010). Haptic and sound interface for shape rendering. Presence Teleoperators Virtual Environ..

[24] Lammertse P., Frederiksen E., Ruiter B. The HapticMaster, a New High-Performance Haptic Interface.

[25] Bordegoni M., Ferrise F., Covarrubias M., Antolini M. (2011). Geodesic spline interface for haptic curve rendering. IEEE Trans. Haptics.

[26] SATIN Project. http://www.youtube.com/user/SATINproject.

[27] Strain Gages, HBM. http://www.hbm.com/.

[28] Lighting Stripes Device. http://www.elshine.it.

[29] Hassenzahl M., Tractinsky N. (2006). User experience-a research agenda. Behav. Inf. Technol..

[30] Covarrubias M., Antolini M., Bordegoni M., Cugini U. A Spline-like Haptic Tool for Exploration and Modification of Digital Models with Aesthetic Value.

[31] Alonso-Arevalo M.A., Shelley S., Hermes D., Hollowood J., Pettitt M., Sharples S., Kohlrausch A. (2012). Curve shape and curvature perception through interactive sonification. ACM Trans. Appl. Perception (TAP).

[32] Covarrubias M., Bordegoni M., Cugini U. (2013). Continuous surface rendering, passing from CAD to physical representation. Int. J. Adv. Robot. Syst..

